# Causal Pluralism in Medicine and its Implications for Clinical Practice

**DOI:** 10.1007/s10838-023-09658-1

**Published:** 2023-11-03

**Authors:** Mariusz Maziarz

**Affiliations:** Interdisciplinary Centre for Ethics and Doctoral School in the Humanities, Jagiellonian University, Grodzka 52, 31-044 Kraków, Poland

**Keywords:** Causal pluralism, Causal inference, Philosophy of medicine, RCT, Clinical practice

## Abstract

The existing philosophical views on what is the meaning of causality adequate to medicine are vastly divided. We approach this question and offer two arguments in favor of pluralism regarding concepts of causality. First, we analyze the three main types of research designs (randomized-controlled trials, observational epidemiology and laboratory research). We argue, using examples, that they allow for making causal conclusions that are best understood differently in each case (in agreement with a version of manipulationist, probabilistic and mechanistic definitions, respectively). Second, we analyze clinical practice and argue that these manipulationist, probabilistic and mechanistic causal claims can be used as evidence for different therapeutic decisions. We differentiate among ‘predicting’ that does not change the relata of causal claims, (mechanistic) ‘interferences’, and ‘interventions’ in the strict sense that act on causes to change effects. The central conclusion is that causal claims agreeing with diverse concepts of causality can deliver evidence for different types of therapeutic decisions.

## Introduction

Obtaining causal knowledge is a major concern for biomedical sciences. Still, there is little agreement among philosophers and in-house methodologists about the concept of causality adequate to the research practices in the discipline. Some pointed to a version of the regularity or probabilistic theory of causality. According to Karhausen (2000), cohort and case–control studies presuppose that causes are, respectively, sufficient and necessary conditions. Russo (2009) interpreted both case–control and cohort studies as presupposing a ‘variational’ view on causality, i.e., delivering difference-making evidence. Thygesen, Andersen, and Andersen (2005) argued that Hill’s (1965) causal criteria overlap with regularity and manipulationist (labeled generative) views. Charlton (1996) supported the view that epidemiological causes are necessary conditions. Recently, Gillies (2018) argued that a probabilistic formulation of Menzies' and Price’s (1993) agency theory is adequate to how causal relations are understood in medicine. Finally, based on the case of exposome research, Canali (2019) supported evidential pluralism.

This question can also be tackled from a normative angle. For example, Weed (1997) opposed the use of Hill’s (1965) causal criteria on the grounds that they limit the scope of research. Russo and Williamson (2007; 2011) advised the health sciences to make causal claims based on both mechanistic and correlational (difference-making) evidence to establish causality. This view motivated the emergence of the evidence-based medicine plus (EBM +) movement (see Parkkinen et al. 2018) that opposes the evidence-based medicine (EBM) leaning towards the difference-making evidence and advised putting equal weight to the results of correlational and mechanistic studies (Clarke et al. 2013). Recently, Williamson (2019) argued that the Russo-Williamson Thesis is superior to the EBM movement in delivering an adequate definition of causality because it not only accounts for research practice, which involves mechanistic reasoning but also offers a theoretical underpinning to extrapolation.

Motivated by all the arguments pointing at different views on causality, we support causal pluralism as the concept of causality adequate to research practices in medicine and explore some implications for therapeutic decisions in the clinic. In most basic terms, causal pluralism is the view that there is “no single thing that is picked out by causality” (Williamson 2006, 72). Each of the multitudes of monistic accounts of causality has faced severe criticisms and counterexamples (Williamson 2006), and, as Illari and Russo (2014, 249) put it, “pluralism may be suggested as the solution: you don’t have to choose *one* account, just take them all, or a carefully selected subset of accounts, to handle all the causal situations you need to think about”.

But causal pluralism is itself a pluralistic position. One can be pluralist about the ontology of causation and claim that there are various types of causal relations in the world. An alternative would be to focus on epistemology and argue that alternative ways of justifying causal claims are warranted or be pluralist about the concepts of a cause that are used for distinguishing causal and non-causal relations (see Illari and Russo 2014, 250–252; Godfrey-Smith 2009, 326–337; De Vreese 2008). Still another version of pluralism focuses on the types of evidence. Notably, Russo & Williamson (2007; 2011) argued that establishing causality in the health sciences requires both difference-making and mechanistic evidence, i.e., it requires knowing that interventions on a cause change its effect and understanding how they do so.

The views of ontological causal pluralists have in common emerging from the counterexamples showing that none of the monistic accounts describe all relations deemed causal, but they are differentiated concerning the number and types of causal relations. Hall distinguished causes making a difference to their effects and producing effects. Skyrms (1984, 245–255) argued that causation in the physical world is a mix of three concepts (statistical causation, transfer of energy, and manipulability) that operate jointly in some contexts but not in others. Instead, Anscombe (1975) and Cartwright (2004; 2007) argued that each causal-family word such as ‘push’, ‘produce’, ‘lower’, etc. denotes a different type of causal relation since “causal arrangements of the world may be indefinitely variable” (Cartwright 2004, 818).

A different version of pluralism is to focus on the concept of causality. Godfrey-Smith (2009) defined pluralism about the concept of cause as a position claiming that C causes E iff the relation satisfied the ‘contextually appropriate’ definition of causality. Psillos interpreted the main five approaches to causation in terms of symptoms of causality instead of ontological accounts and compared causation to the common cold. He argued that both are “a rather loose condition with no single underlying nature” (Psillos 2010, 133). Psillos distinguished agnostic and atheistic versions of conceptual pluralism about causality and claimed that the former, remaining silent about the nature of causation, is a safer view but defended the latter. We, instead, defend pluralism about the concept of ‘cause’ in medicine and remain agnostic about ontology.

Pluralism about the concept of cause has been recently supported in philosophy of medicine. Campaner and Galavotti (2012; 2007) distinguished between manipulative and mechanistic evidence and argued that there are, in parallel, two distinct concepts of ‘cause’ in the health sciences, and some causal inferences and therapeutic decisions rely solely on manipulative evidence stemming from experimentation and observation without an understanding of the mechanism of treatment’s action. llari and Russo (2014) supported pluralism not only about the concept of cause but also purposes of causal inference and philosophical questions. They argued that “[w]orldly causes seem to be very different; we also have different sources of evidence; and the methods of the sciences themselves are also pretty diverse. What form of pluralism suits a given scientific context is at least partly an empirical question. We need to study the field, its particular problems and methods, and work out what tiles and what fragments we need, so that we can start to build up a [causal] mosaic” (Illari and Russo 2014, 259; see also Russo 2022, 98–113).

Vandenbroucke et al. (2016) and Broadbent et al. (2016) have argued for the position of pragmatic pluralism as a solution to the problem that the dominant approach to causal inference, the Restricted Potential Outcome Approach narrows down both the repertoire of causes and of research methods used in epidemiology. It is so because RPOA focuses on humanly made interventions and assesses the usefulness of causal claims based on the possibility of predicting the effects of hypothetical interventions. Vandenbroucke et al. (2016) criticized RPOA because it focuses on a single study design instead of addressing the question of how to amalgamate evidence stemming from different types of evidence. In their support for pragmatic pluralism about the concept of causality, Vandenbroucke et al. (2016, 1784) argued that “[e]pidemiologists should recognize that there are different ways of thinking about causality, and they should use the approach that seems most apt for the epidemiological problem at hand”.

In response to their paper, Weed (2016) harshly attacked the supporters of pragmatic pluralism by claiming that “it is not sufficient to simply critique a method (or an approach such as RPOA), identify its problems (even errors) and call it a day. To make progress (i.e., to make improvements), an alternative must be proposed that does at least several things: it corrects the errors in the original method, it keeps the features of the original method that ‘work’ and it does not add new problems (errors) that make it less desirable than the original method”. (Weed 2016, 1839). Weed (2016) criticized Vanderbroucke et al. (2016) for offering a proposal based on causal pluralism that is too vague to be introduced to research practice and too reliable on the vague notion of judgment. While his criticism may be an exaggeration in the sense that replacing a misguided approach with an adequate but vague one may still be beneficial, our argumentation might be considered as a development of the proposal of Vandenbroucke et al. (2016).

The stance of pragmatic causal pluralism defended by Vandenbroucke et al. (2016) acknowledges that using the context-dependent notion of ‘cause’ is beneficial for clinical practice. Both Vandenbroucke et al. (2016), Broadbent et al. (2016) and this paper highlight the usefulness of evidence stemming from randomized clinical trials and observational studies in humans and laboratory research. We agree that none of the monistic accounts of causality (including a version of the manupulationist account underlying RPOA) can encompass all inferences and support the pluralism about the concept of causality while remaining silent about ontology and develop pragmatic pluralism by showing that three notions of causality are involved in causal inferences in medicine and show how these causal conclusions agreeing presupposing different causal concepts warrant alternative types of therapeutic decisions. In particular, we develop their analysis by delineating the exact meaning of the pluralist stance and claiming that different types of evidence warrant alternative types of intervening in the world so that evidence stemming from various or top-rated sources is not always needed. In our view, the concept of causality adequate to medicine includes a version of the probabilistic, mechanistic and manipulationist account of causality. Additionally, we analyze how the evidence sufficient for supporting the probabilistic, mechanistic and manipulationist causal claims suffices for undertaking various decisions in the clinic.

In Section 2, we analyze the three main research designs in medicine (observational studies, laboratory research and interventional clinical trials) and argue that causal claims based on these types of research are best understood as agreeing with probabilistic, mechanistic and manipulationist views on causality. This deems causal pluralism adequate to the whole spectrum of causal inferences in medicine. In Section 3, we investigate the implications of causal pluralism for clinical practice, analyze the notion of therapeutic decision, and argue that it encompasses different types of interventions. Furthermore, we make the point that probabilistic, mechanistic and manipulationist causal claims are evidence sufficient for, respectively, ‘predictions’, ‘mechanistic interferences’, and ‘narrowly-construed interventions’. Finally, we conclude that the conclusions established based on RCTs, observational epidemiological studies, and laboratory research agree with the manipulationist probabilistic and mechanistic views on causality. These differently understood causal claims deliver evidence for alternative types of clinical decisions.

## Causal Pluralism in Biomedicine

Biomedicine is an extensive discipline that employs starkly differentiated research methods. They include basic research (in silico simulation, in vitro and in vivo research involving studies on cell cultures and animal models), non-interventional studies (case reports and case series, cross-sectional, case–control and cohort/follow-up studies), and interventional clinical trials (pre-post design, nonrandomized and randomized controlled trials) (Murad et al. 2016; OCEBM Levels of Evidence Working Group 2011). The movement of evidence-based medicine that prioritizes RCTs over non-interventional studies and in-vitro research based on the risk of confounding characteristic for each type of study (Borgerson 2009; La Caze 2009) seems to interpret all research methods as delivering difference-making evidence or evidence for manipulationist causality. Another stance is to acknowledge that the differentiation of research methods suggests that they provide different types of evidence but remain monistic or agnostic concerning the concept of causality. The EBM + movement accepts that the evidence delivered by different research methods differs in quality and some types of evidence are more suitable for inferring mechanisms while others for supporting difference-making conclusions (Parkkinen et al. 2018). This position stems from the normative reading of the Russo-Williamson Thesis (Russo and Williamson 2007). As rightly observed by Broadbent (2011), the thesis that causal claims require difference-making and mechanistic evidence (see Illari 2011) can be interpreted as “a descriptive account of causal inference […] or as a normative account of the standards which ought to be used when deciding whether to infer causation” (Broadbent 2011, 58).

Still another possibility is to be pluralist about concepts of causality. In that case, one endorses the view that the evidence delivered by different research methods suffices for establishing various kinds of causality (see Reiss 2015) or relations deemed causal by different concepts of causality. Suppose laboratory research, observational and interventional studies deliver evidence sufficient for inferring relations that are deemed causal by different concepts of causality. Three views on causality (probabilistic, mechanistic and manipulationist) are particularly relevant to the health sciences.

The probabilistic view dates back to work, which claimed that causes change the probability of their effects and precede them in time. Wiener (1956) identified causality with predictability in the context of time series (accordingly, variable $$X$$ causes variable $$Y$$ if knowing the history of $$X$$ improves the accuracy of predictions of $$Y$$ based on its history). Granger (1969) directly applied this view to econometric tests for Granger-causality among variables. They are now entering the toolbox of data-intensive natural sciences such as neuroscience and ecology Suppes (1970). Generalized Hume’s (1956 [1739], 170) regularity definition into the indeterministic environment and distinguished between the *prima facie* $$$$P\left(Y|X\right)>P\left(Y\right)$$$$ and fully-fledged condition $$P\left(Y|X\Omega \right)>P\left(Y|\Omega \right)$$, where $$X$$ and $$Y$$ stand for the relata of a causal claim and $$\Omega$$ denotes all relevant factors. The prima-facie definitions voiced by Suppes (1970) and Granger (1969) are easily applicable, but they are susceptible to the problem of confounding, where some other factor produces observed correlation. In contrast, the fully-fledged conception that requires the knowledge of all other factors influencing the probability of an event *C* (in Suppes’ formulation) or all history of the universe (in Granger’s formulation) cannot be directly applied to causal inference. Given the concern of biomedical researchers with token-level causality, a definition of causality with variables instead of events as relata seems a good candidate (see Hausman 2005). Therefore, the probabilistic concept of causality applicable to research can be formulated as follows: *X* causes *Y* iff the conditional distributions of *Y* differ for different values of *X* and (values of) *X* precede (related values of) *Y* in time, given some background conditions *C*, where *X*, *Y,* and *C* are variables and *C* are (some) other causes of *Y*.

Facing the problems of common causes and spurious correlations (see Sober 2001), some philosophers argued that what distinguishes causal from accidental relations is that a causal mechanism produces the former. There is considerable disagreement about what precisely the mechanisms are. Machamer et al. (2000, 3) put forward a (minimal) definition of mechanisms as “entities and activities organized such that they are productive of regular changes from start or set-up to finish or termination conditions”. Illari and Williamson (2012, 119) argued that, across scientific disciplines, mechanisms are “entities and activities organized in such a way that they are responsible for the phenomenon”. The gist of these and other definitions put forth by New Mechanists is that actions and interactions of some entities produce causal relations, and mechanisms consist of these entities and their actions and interactions (Glennan 2017; Glennan and Illari 2018). According to the mechanistic view: *X* causes *Y* iff there is a mechanism by which X contributes to the production of Y.

Finally, causality has been defined as relations invariant under intervention. While the manipulationist approach (which includes interventionist and agency theories) has originated as a response to neopositivist reductionism (Collingwood 2001 [1940]), later theories reduce causal relations to a more primitive notion of human agency (Menzies and Price 1993). According to agency theories, the ability to bring about *Y* by changing/producing *X* is considered easier to grasp for humans in comparison to the philosophical notion of cause. Causal relations can also be defined as relations invariant under theoretically-defined (atomic) interventions (e.g., Woodward 2003) that refrain from using the notion of human agency due to some philosophical problems. Such interventions *I* on *X* are required to be statistically independent of any other variables in a causal model, set the value of *X* (so that any other factor does not influence it), and not change *Y* by the way other than through *X*. Given that such ideal interventions could only be introduced by an ideal RCT and not actual clinical trials (see Reiss 2019, 3113–3114; Gillies 2018, App. 1), the definition being a gist of the agency theories seems a better candidate for the concept of causality relevant for medical research and practice. Hence, according to the manipulationist view, *X* causes *Y* iff the relation between *X* and *Y* can be used for effective interventions, i.e., a change of *X* brings about a change in (the probability of) *Y*.

Below, we argue that causal pluralism (here understood as encompassing the three concepts of causality) is the view on causality adequate to the whole spectrum of research practices in medicine. Probabilistic, mechanistic and manipulationist views on causality best describe the types of relations that can be discovered by, respectively, observational studies, laboratory research and interventional clinical studies. First, we argue that the conclusions of observational studies are best understood as probabilistic causal claims because the risk of confounding (common causes) is higher than in (randomized) interventional studies. Second, in agreement with the EBM + movement, we interpret laboratory research as delivering mechanistic evidence warranting mechanistic causal claims. Finally, we consider an RCT aimed at assessing a treatment’s side effects and argue that the conclusions based on RCTs are best understood as manipulationist causal claims.

### Correlational Evidence

Epidemiology, here narrowly construed as a subdiscipline dealing with observational data and contrasted with interventional trials discussed in Section 2.3., is usually defined as a field striving for identifying and measuring the impact of biological, socioeconomic and behavioral causes of disease and health (Rothman and Greenland 2005; Galea et al. 2010). The focus on causal inference is sometimes hidden behind the discussion of associations, risks and correlations because causal inferences from observational data are frowned upon by some journal editors and epidemiologists aware of the risk of confounding (usually denoted by philosophers as a common-cause fallacy) (Hernan 2018). Despite no explicit claims in some cases, Broadbent (2021) asserted in his response to Hernan (2018, 4) that “observational epidemiological studies aim to attain causal knowledge”. The strive for explicitly causal conclusions is further supported by Jukola’s (2019) argument that evidentiary standards for establishing causality should be adjusted when interventional studies are not feasible. This view, if generalized and applied to the whole area of observational epidemiology, poses the question of whether the concept of causality used to distinguish causal and non-causal results of observational studies should be the same or different from the one used in causal inferences from randomized controlled trials (see De Vreese 2009).

Studies of risk factors of heritable diseases are one of the areas of medicine where conducting RCTs is unethical and infeasible. For this reason, difference-making evidence can only come from observational studies. For example, the risk factors of autism spectrum disorder (ASD) remain uncertain (Happé et al. 2006). A few epidemiological studies suggest the influence of paternal age on ASD. Still, most studies are of lower quality (utilizing the case–control instead of cohort design) and do not control for confounding (Reichenberg et al. 2006, 1027).

Furthermore, the three existing studies that control for maternal age deliver conflicting results (Burd et al. 1999; Larsson et al. 2005; Glasson et al. 2004). The presence of conflicting epidemiological results regarding the influence of maternal age on the risk of ASD in their children suggests that the relationship between maternal age and a child’s risk of ASD is possibly confounded. Given that paternal age is known to be positively related to the risk of psychiatric disorders in children, Reichenberg et al. (2006, 1027) hypothesized that “advancing paternal age is associated with increased risk of ASD in offspring”. Their study is an excellent example for scrutinizing here because it is representative of a broader spectrum of methods used in observational epidemiology. Reichenberg et al. (2006) conducted a cohort study of all adults born in Israel in six consecutive years of the 1980s based on data obtained from health checks prior to compulsory military service. Their conclusion that paternal age is a risk factor for ASD is substantiated primarily by a logistic regression (labeled ‘full model’ henceforth) that estimates the influence of paternal age on the probability of child’s ASD controlling for year of birth, maternal age and socioeconomic status (Reichenberg et al. 2006, 1028):

$$logit\left(p\right)={log}_{e}\left(\frac{p\,({ASD}_{n}=1)}{p\,({ASD}_{n}=0)}\right)={\alpha }_{0}+{\alpha }_{1}{P}_{n}+{\alpha }_{2}{Y}_{n}+{\alpha }_{3}{S}_{n}+{\alpha }_{4}{M}_{n}$$

where: $p\,({ASD}_{n}=1)$—the probability that child *n* suffers from ASD;

${P}_{n}$—paternal age at birth of the *n-*th child;

${Y}_{n}$—year of birth for the *n-*th child;

${S}_{n}$—socioeconomic status of the *n*-th child;

${M}_{n}$—maternal age at birth for the *n*-th child; ${\alpha }_{x}$–the estimated parameters.

Furthermore, the researchers estimated a simplified version of the model that measures the influence of paternal age on the risk of ASD in offspring without controlling for other factors:

$$logit\left(p\right)=\left(\frac{p\,({ASD}_{n}=1)}{p\,({ASD}_{n}=0)}\right)={\alpha }_{0}+{\alpha }_{1}{P}_{n}$$

Reichenberg et al. (2006, 1028) used Wald ${\chi }^{2}$ two-sided test to check the significance of obtained estimates. The estimations of all parameters proved to be statistically significant. The two regressions show that there is a positive association between father’s age at the birth of his children and their chance of developing ASD (i.e., α_1_> 0).

At the time of the publication of Reichenberg et al.’s (2006) study, there had been no reliable mechanistic evidence. Ratajczak (2011) and Miles (2011) listed genetic causes, pollution during pregnancy and social factors. Additionally, Reichenberg et al. (2006) hypothesized two possible genetic mechanisms (imprinting and mutagenesis) linking paternal age to the risk of ASD, but these should be regarded as hypotheses for further research instead of plausible mechanistic theories since no empirical evidence supports them. The distinction between hypothesizing that a mechanism operates and delivering evidence for that mechanism is crucial and seems not to be recognized by those who interpret statistical modeling as providing both difference-making and mechanistic evidence. While statisticians consider data-generating processes that underlie statistical models, such models neither deliver evidence for the processes nor describe it in any detail. In particular, statistical models do not inform about the entities and their interactions but only estimate aggregate-level dependencies. Identifying entities of a mechanism and their interactions and delivering some support for it seems to be the necessary condition for claiming that a study provides evidence for mechanisms that could be used in support of a causal claim. Furthermore, the data-generating process underlying a statistical model can be false if a model does not include a confounding factor or includes spurious correlations to improve its predictive power. For these reasons, formulating a causal conclusion based on a statistical analysis of observational data is not warranted if one is committed to the mechanistic view on causality.

Given this, the remaining plausible concepts of causality are different versions of probabilistic and manipulationist views. Reichenberg et al. (2006) established the causal claim based on the presence of a partial correlation between paternal age and the risk of ASD and time precedence. As La Caze and Winckel (2020) observed, such practice is widespread and causal claims are often established based on difference-making evidence alone in cases when it is of sufficient quality. Even though the concept of difference-making evidence (as construed by the program of EBM +) encapsulates both correlational evidence from observational studies and manipulationist evidence from RCTs (Illari 2011; Russo and Williamson 2011), recognizing the distinction between the two is crucial for understanding how causal conclusions established based on observational studies can be interpreted.

Despite the difference between observational and interventional studies is often considered one of degree in epidemiology (e.g., Hernán 2018), cohort studies, being studies of observational data, deliver only weak evidence for the invariance of a relation under intervention. It is so not because randomized studies have well-defined interventions as argued for by Hernán and Taubman (2008), but because the possibility of confounding cannot, in principle, be excluded. Even if one controls for all known confounders in an observational study, it is possible that some *unknown* confounders remain imbalanced across the treatment and control groups and this imbalance may have an effect on the effect size estimate. Statistical models can establish causality in *x*-dimensional model space. Still, clinical decisions are undertaken in a more (*x* + *n*-)-dimensional world space, and therefore statistical dependencies may turned out not to be causal in the manipulationist sense when other (possibly unobservable) variables are included in the model. This has been established for Granger-causality tests (Lütkepohl 1982), but the argument holds for other statistical models of observational data in macroeconometrics (Henschen 2018) and equally well applies to observational epidemiology. As Broadbent (2021, 2625) put it, “[r]esidual confounding is an ineliminable risk of observational studies”.

Randomization leading accidently to unbalanced confounding makes confounding trouble also RCTs. However, the benefit of the randomized assignment is that it allows for calculating the probability of baseline imbalances. Statistical hypothesis testing, which is a standard approach to analyzing data from RCTs, assert that the probability of baseline imbalance is lower than the threshold for statistical significance (ALPHA =  0.05, usually). For this reason, observational studies do not warrant conclusions in line with the manipulationist view on causality and the difference between the two types of studies is substantial rather than one of degree. In the case of observational studies, researchers cannot include unknown or unmeasurable confounders and need to choose only the most relevant known factors to keep the number of explanatory variables sufficiently low. In contrast, proper randomization statistically controls for both known and unknown confounders (see Cartwright 2010; La Caze 2009). For these reasons, Illari (2011, 143) contended that “[t]he problem [of confounding] is of course also present to a much greater degree in other kinds of studies, such as observational studies”.

What follows, the claim of Reichenberg et al. (2006) could be interpreted in line with the manipulationist view in a four-dimensional (model) world. However, paternal age is possibly only a proxy for the actual cause(s) of ASD (e.g., the number of mutations in specific alleles). Therefore, taking this evidence as support for the claim that changing paternal age (in a more dimensional world) leads to reducing ASD risk in offspring is not warranted. If there is another factor (e.g., the number of genetic mutations in specific alleles), having children earlier in life will not reduce the risk of ASD. Hence, the result of Reichenberg et al. (2006) and other studies belonging to observational epidemiology is best interpreted causally in agreement with a version of the probabilistic view on causality. In particular, their simplified model can be shown to be a direct application of Suppes’s (1970) token-level *prima facie* definition of causality generalized into the type-level context of variables (instead of events). According to this definition, *X* causes *Y* iff the conditional distributions of *Y* differ for different values of *X* and (values of) *X* precede (related values of) *Y* in time. The definition of causality presupposed in the full model, by contrast, is located between the *prima facie* and the fully-fledged concept because the model controls for some (but likely not all) confounders: variable *X* causes variable *Y* iff the conditional distributions of variable *Y* differ for different values of *X* given some background conditions and the values of *X* precede values of *Y* in time.

### Mechanistic Evidence

Evidence for mechanisms comes primarily from experimental manipulation in target or surrogate model, observation and analogical reasoning from other, already known mechanisms (Parkkinen et al. 2018, 78). While RCTs and observational studies can suggest that a causal mechanism is present, laboratory research (e.g., in vitro and animal studies), biomedical imaging and analyzing tissue samples are particularly useful for discovering the entities and activities that constitute that mechanism and characterize it in some detail (Aronson 2020). The results of such studies are not trustworthy for predicting the efficacy of interventions (GRADE Working Group 2016; OCEBM Levels of Evidence Working Group 2011) because the operation of one mechanism can be stopped or screened off by some other causal factor and the problem of extrapolation (Steel 2007; Howick et al. 2013) but mechanistic evidence is considered to play a significant role in corroborating the results of correlational studies (Russo & Williamson 2007; 2011; Parkkinen et al. 2018).

In contrast to the methodological advice stemming from the normative reading of the Russo-Williamson Thesis, in some cases, biomedical researchers make causal claims based on mechanistic evidence alone. Recently, the news that blue light emitted by electronic devices causes blindness hit the headlines worldwide (Dodgson 2018). While blue light has been sometimes considered a risk factor for age-related macular degeneration (AMD) and cataracts (Taylor et al. 1990), epidemiological results are conflicting, and hence reliable difference-making evidence for the claim that blue light causes harm to human eyes is missing. It was previously hypothesized that blue light exposure is a risk factor for AMD due to oxidative stress (Fletcher 2010). In contrast, Ratnayake et al. (2018) obtained evidence that suggests that a different factor plays a causal role and discovered the mechanism that underlies the dependency. According to their results, all-trans-retinal (ATR) present in photoreceptors, when exposed to blue light, distorts one of the phospholipid components of the cellular membrane, PIP_2_. This disrupts the functioning of cells and results in their death. Ratnayake et al. (2018) discovered the toxicity of blue light using in vitro experiments. Their research design involved the introduction of retinal into different types of cell lines (tumors, animal and human cells) and exposing them to blue light. Both the duration of exposure and power of light differed across experiments. Ratnayake et al. (2018) used, as controls, cell lines without retinal and those cell lines that included (artificially introduced) retinal but were not exposed to the potentially phototoxic light source. The results were observed using different biochemical methods aimed at analyzing the products of the photochemical reactions induced by blue light.

The explanation for the phototoxicity of blue light to human eyes delivered by Ratnayake et al. (2018) is of mechanistic nature. First, the description consists of different entities (photons, retinal and other cellular compounds). Second, the laboratory research uncovers how these entities act and interact with each other and how these actions and interactions produce a higher-level causal relation between blue light-exposed cells including retinal and their death. Finally, Ratnayake et al. (2018) refer to their results using the notion of mechanism (e.g., Ratnayake et al., 5). The lack of trustworthy difference-making evidence further supports this interpretation. Neither RCTs nor cohort studies are feasible because AMD develops late in life, and measuring sun or blue light exposure reliably is challenging. Case–control studies, which are usually considered to deliver evidence of mediocre quality, provide conflicting results. Some studies find sunlight exposure protective, while others report negative effects (West and Schein 2005) and for this reason the difference-making evidence (i.e., evidence for blue light exposure being correlated to age-related macular degeneration) stemming from human studies was missing.

But an opponent disagreeing with the claim that Ratnayake et al. (2018) draw the causal conclusion based on mechanistic evidence alone could say that the laboratory research involves conducting manipulations to the experimental setting and observing differences between the intervention and control groups even if the instances assigned to those groups are cells or cellular structures (organelle) and for this reason such a study delivers not only mechanistic evidence (theoretical understanding of how blue light of a particular wavelength makes retinal cells die) but also difference-making evidence, showing that the intervention (exposing cells including all-trans retinal (ATR) to the blue light) correlates to cellular death. Whilst such an argument seems plausible at first, it is in fact fallacious for the reason that the laboratory in vitro research involves experimenting on cells (as in this case) or cellular structures. Consequently, such studies can only deliver difference-making evidence concerned with what happens in vitro. Such studies are in principle unable to prove that an intervention studied in a laboratory is effective in vivo due to the problem of masking or, more broadly, mechanism-mechanism interactions. For this reason, despite we wholeheartedly agree with Illari’s (2011, 143) assertion that “the same experimental methods are frequently used to gain evidence of mechanism and evidence of difference-making” (see also Williamson 2019), this can only be said about experimental methods conducted on humans (clinical trials) unless one is interested in a causal claim concerned with causality in cell lineages.

To put it differently, the study of Ratnayake et al. (2018) produced also difference-making evidence that exposing cells including ATR to blue light kills them in vitro but it does not support claiming that exposing humans (their eyes) to blue light makes them develop age-related macular degeneration. Ratnayake et al. (2018) have not used the evidence for regularity from their in vitro studies to substantiate their results because the regularity observed in vitro can only warrant establishing a probabilistic or manipulationist causal claim concerned with what happens in the laboratory. In contrast, the interest of Ratnayake et al. (2018) in establishing causality in humans is visible when they analyze the influence of household fluorescent and LED light on cellular death.

Therefore, their causal conclusion concerned with the influence of blue light on developing AMD in humans must be based on the mechanistic evidence described in the paper. The in vitro research conducted by Ratnayake et al. (2018) allows for discovering neither empirical regularity (probabilistic dependence at the aggregate level of organisms) nor relations invariant under intervention. This research allows for discovering regularities, but only at the level of interactions among entities that constitute the mechanism. Evidence for the mechanism is insufficient for concluding that exposure to blue light produces a clinically relevant (or even observable) effect in humans. It does not allow claiming that exposing human eyes to blue light leads to blindness (neither deterministically nor in most cases). The reason is that other mechanisms operating in the world may prevent this one from producing the effect that appears under experimental closure. What follows, a newly discovered mechanism cannot be considered difference-making evidence for blue light having an obserable effect on age-related macular degeneration in humans. As Russo and Williamson (2007, 162) put it, “mechanistic evidence on its own cannot warrant a causal claim, as it may be the case that the purported cause […] actually makes little or no difference to it.” Therefore, the causal conclusion of Ratnayake et al. (2018) and other researchers that make causal claims based on laboratory research is best interpreted in terms of a version of the mechanistic concept of causality: *X* causes *Y* iff there is a mechanism by which X contributes to the production of Y.

### Manipulationist Evidence

Randomized controlled trials (RCTs) allow for estimating the average treatment effect (ATE) that approximates the average effect of an intervention (treatment) in a sample because the random assignment to treatment and control groups asserts, at least in principle, equal distribution of unknown confounders (Cartwright 2010; Worrall 2002, 321–328; see Deaton and Cartwright 2018). Most RCTs aimed at assessing the effectiveness of new therapies are started only after researchers gather evidence sufficient to expect the treatments to be successful. This practice can be explained by the high costs of conducting clinical trials and the ethical concerns related to the well-being of participants. Therefore, RCTs usually test compounds that are also supported by mechanistic evidence stemming from laboratory research.

For this reason, most efficacy claims in medicine can be interpreted in agreement with evidential pluralism. In contrast, the trials assessing adverse drug reactions usually lack mechanistic evidence. Such practice is an effect of the view that the nonepistemic values related to safety concerns outweigh the risk of drawing causal conclusions based on difference-making evidence alone. One such example is the leg of the RECORD study aimed at assessing the side effects of rosiglitazone. Despite the criticism highlighting the use of a biased sample and inappropriate inclusion criteria (Stegenga 2018, 143), the study serves as an excellent example of an RCT conducted without prior mechanistic knowledge.

Home et al. (2007) evaluated the risk of cardiovascular attacks among patients treated with rosiglitazone compared to other diabetes drugs. Over 4.000 patients experiencing poor control over diabetes treated with either sulfonylurea or metformin were randomly assigned to the treatment and control groups. Patients allocated to the control group received both drugs (sulfonylurea + metformin) while the treatment group received rosiglitazone as a second drug (sulfonylurea or metformin + rosiglitazone). To assess the safety of rosiglitazone, Home et al. (2007) compared the number of hospitalizations and deaths resulting from all cardiovascular causes. The differences between the treatment and control groups were statistically insignificant except for deaths caused by congestive heart failure (CHF). Later research showed the inferiority of the rosiglitazone treatment (Hiatt et al. 2013), and therefore we focus on this secondary outcome instead of the overall comparison of deaths and hospitalizations caused by all cardiovascular causes. Given the random allocation of patients among treatment and control groups that statistically (in the long run) asserts the equal distribution of confounding factors among the treatment and control groups, the difference in the prevalence of CHF can be ascribed to the intervention, i.e., the rosiglitazone therapy. Therefore, the RECORD study demonstrates that treating diabetes patients with either sulfonylurea or metformin and rosiglitazone instead of sulfonylurea and metformin raises the number of CHF incidents in the treatment group. That is, the change in the prevalence of CHF can be ascribed to the experimental intervention.

Furthermore, at the time of the RECORD study, the claim that rosiglitazone raises the prevalence of CHF had not received support from evidence other than RCT. In particular, Nissen and Wolski (2007), who shed light on the potentially harmful effects of rosiglitazone concerning cardiovascular disease with their meta-analysis, admitted that the mechanism of action was poorly understood. “PPAR agonists such as rosiglitazone have very complex biologic effects, resulting from the activation or suppression of dozens of genes. The patterns of gene activation or suppression differ substantially among various PPAR agonists, even within closely related compounds. The biologic effects of the protein targets for most of the genes influenced by PPAR agonists remain largely unknown.” (Nissen and Wolski 2007, 2465). Considering that ‘dozens of genes’ are involved in the pharmacological effects of PPAR agonists (some of which are activated while others suppressed, with PPARα alfa, PPARδ and PPARγ showing affinity to different receptors), the RCT (as conducted by Home et al. 2007) cannot be said to deliver mechanistic evidence for a specific mechanism (among several possible pharmacological effects).

Even though some (specifically designed) RCTs can deliver mechanistic evidence (evidence for a mechanism), the main advantage of randomization is that RCTs can serve as a black-box tool (Howick 2011) that supports causal conclusions even if the details of how an intervention works remain unknown. It is so because randomization, in principle or ideal RCTs (see Cartwright 2007), asserts the equal distribution of confounding factors across the treatment and control groups. In well-conducted (actual) RCTs, randomization asserts that neither confounding nor chance are likely to account for the difference between treatment and control groups (Illari 2011; La Caze 2009), and therefore the difference between treatment and control groups can be interpreted as resulting from the intervention.

Marchionni and Reijula (2019) argued that properly designed laboratory experiments in economics (resembling to some degree clinical trials) allow for discriminating between two plausible mechanisms producing a phenomenon and hence deliver evidence for one of them. The same can be said about RCTs in medicine. For example, one could conduct an RCT to test the mechanism of interaction between temozolomide (a drug developed for glioblastoma, a brain tumor) and the MGMT gene. If the RCT would confirm the observational result of Hegi et al. (2005) that patients with the gene do not respond to therapy, then it would support our current (mechanistic) understanding of how the gene codes a protein that counteracts the mechanism of the drug’s action. However, most RCTs assessing efficacy rely on some mechanistic evidence (evidence from mechanisms or mechanistic reasoning) (see Rocca 2018), but observing the difference between treatment and control groups delivers difference-making evidence. The mechanistic evidence understood as features of specific mechanisms, which enter RCTs at the planning stage, emerges from basic science research (La Caze 2011) such as in vitro and in vivo experiments, observation technologies and analogy (Parkkinen et al. 2018, 78). For this reason, the best example of how RCTs work in the context of causal inference are studies assessing adverse drug reactions because there is usually limited or no mechanistic evidence for a treatment’s side effects (see Sect. 2.3.)

Another take on how RCTs could deliver mechanistic evidence is to argue that by demonstrating the link between intervention and outcome, RCTs provide evidence that there is some mechanism linking the two. There are two problems with this reasoning. First, as Howick (2011, 34) observed, it is circular due to taking the observation of difference-making as a sign of an underlying mechanism. The reason is that accepting the mechanistic ontology equates to acknowledging that mechanisms produce causal relations, and observing such relations is a sign of an underlying mechanism. Second, even if the problem of circularity could be ignored, RCTs (with some exceptions considered above) neither depict a hypothesized underlying mechanism nor deliver evidence for the hypothesized description thereof. Referring to the views on what are the sources of evidence for mechanisms in medicine supports this view. Illari (2011; 2016) defined such evidence as the technologies that allow for studying entities constituting a mechanism and their interactions, particularly studies that help identify or better understand entities, their activities and organization. Also, Parkkinen et al. (2018) do not include RCTs in research designs that deliver mechanistic evidence. To reiterate, well-conducted RCTs are very good at showing that intervention under study leads to an observed outcome regardless of the mechanism of action but only indirectly allow for inferring that there is a mechanism linking the intervention with the observed outcome.

Given this, Home et al. (2007) supported their causal conclusion with difference-making evidence only. In contrast to the evidence stemming from observational epidemiology, randomization (jointly with large sample sizes) makes confounding due to known and unknown factors unlikely. Therefore the difference in outcomes (ATE) between treatment and control groups can be ascribed to (is very likely to result from) the experimental intervention. The risk of baseline imbalances created by randomized assignment cannot be excluded, but is very low and described by the level of statistical significance. Given this, the randomized study delivers evidence for a probabilistic causal claim (similarly to observational studies, see Sect. 2.1.) and manipulationist causal claim. In some sense, large randomized studies can also be said to deliver mechanistic evidence as showing that the intervention under study has an effect on an outcome of interest which implies that there is a mechanism linking the two (see Williamson 2019). However, standard RCTs allow for indirectly making inferences about mechanisms unless they are designed in a specific way to test a hypothesis about a mechanism. In particular, the RECORD study did not describe how rosiglitazone influences the probability of congestive heart failure and hence this particular study (and other clinical trials aimed at assessing harms) do not allow for inferring the mechanism of how the intervention has an effect on the outcome. For this reason, interpreting the causal conclusion drawn by Home et al. (2007) in terms of a mechanistic causal claim seems implausible.

The relation discovered by Home et al. (2007) is deemed causal according to both probabilistic and manipulationist views on causality: both conclusions that (1) ‘the probability of CHF is higher in the treatment group (in line with a version of the probabilistic view) and (2) ‘treating patients with rosiglitazone raises the likelihood of CHF’ are warranted. Causal conclusions based on RCTs have previously been interpreted in line with the fully-fledged version of the probabilistic view on causality (see Sect. 2.1.) (e.g., Cartwright 2007) and a version of the manipulationist view (Strand and Parkkinen 2014). However, the risk of confounding in well-conducted RCTs is qualitatively lower than in well-conducted observational studies, making conclusions more likely to remain invariant under intervention. Observational studies can only control for known confounders, as model specification depends on researchers’ understanding of the phenomenon under study. What follows, even though spurious causal inferences from RCTs are in principle possible, they are unlikely (in comparison to the risk of confounding in observational studies). The interpretation in terms of a version of the manipulative causation is further supported by a pragmatic argument: if researchers conducting a study were interested solely in gathering correlational evidence, they would not bother to conduct an interventional study and, to minimize the burden on participants, would proceed with an observational design.

Thus, the RECORD study and other RCTs are best interpreted as a demonstration that the difference between the treatment and control group can be ascribed to the experimental intervention. Given this, a probabilistic formulation of the manipulationist approach such as Menzies and Price’s (1993) theory seems the right candidate (see Gillies 2018). The gist of this view is that *X* causes *Y* iff an agent can influence the probability of *Y* by bringing about *X.*

## The Implications of Causal Pluralism for Evidence-Based Clinical Practice

Above, we have argued that endorsing causal pluralism, understood as encompassing probabilistic, mechanistic and manipulationist concepts of causality, is the stance adequate to the plurality of causal inferences in the discipline. Hereafter, we investigate the implications of causal pluralism for clinical practice. Our argument relies on distinguishing among three types of therapeutic decisions and supports the view that each requires different causal claims. The analysis of the notion of therapeutic intervention justifies the views of EBM supporters on how to assess the quality of evidence and delivers philosophical underpinnings for the differences in quality assessment for different purposes. For example, the Oxford Centre for Evidence-Based Medicine (OCEBM Levels of Evidence Working Group 2011) asserts that systematic reviews of cross-sectional studies are Level-1 evidence for assessing if a diagnostic test is accurate, but efficacy claims require systematic reviews of RCTs or an n-of-1 study.

As we delineate below, these differences can be explained by the distinction between those clinical decisions requiring causal claims invariant under intervention from those that can rely on either observational (probabilistic) or mechanistic conclusions. Given this, the analysis of the notion of clinical decision can lend further support for causal pluralism as the view on causality adequate to research practices in medicine and clinical practice. The movement of evidence-based medicine (EBM) has been concerned with analyzing the “grounds for clinical decision making” (Evidence-Based Medicine Working Group 1992, 2420) since its very beginning. Still, insufficient attention seems to have been paid to the question of what these clinical decisions are. In particular, the notions of ‘interventions’ (e.g., Djulbegovic and Guyatt 2017) and ‘decisions’ (e.g., Coutler, Entwistle and Gilbert 1999) seem to be used interchangeably in an unsystematic way.

The plurality of medical interventions has been recognized, for example, by Krieger and Davey Smith et al. (2016) in their paper supporting the pluralistic methodology of triangulation and inference to the best explanation and opposing the potential outcome approach to causal inference. The epidemiologists motivated the need for conceptual and methodological pluralism in the health sciences by observing that the causal link between adiposity and ischemic heart disease can be influenced not only by pharmacological interventions but also by physiological effects of behavioral changes concerned with diet or exercise. Furthermore, such interventions can be mediated by alternative pathways such as lipid profiles and blood pressure. According to one of the few explicit definitions, “the notion of intervention (e.g., drugs, therapeutic procedures, etc.) encompasses [all] answers to therapy- and diagnosis-related questions” (Lin and Demner-Fushman 2007, 459). Such a broad notion of clinical decisions requires further delineation of what exactly is the subject of these decisions.

The most apparent type of clinical decision, well depicted by Woodward’s (2003) interventionist theory of causality, is to bring about the desired effect by acting on its cause. For example, if one knows that high blood pressure causes strokes, one can lower blood pressure to reduce the risk of a stroke. Such atomic or narrowly-construed interventions require having at hand a manipulationist causal claim that describes a relation invariant under intervention. Neither probabilistic nor mechanistic causal claims are sufficient for these narrowly construed interventions. By delivering evidence for probabilistic causal claims, observational studies are *in principle* susceptible to the common-cause fallacy. Therefore, ‘translating’, (i.e., taking a causal claim presupposing some notion of causality and using it in a way requiring having at hand a causal claim of a different meaning) a causal claim from the probabilistic into manipulationist notion to use it as a ground for intervention may lead to results different from what is expected. Mechanistic causal claims also do not support narrowly-construed interventions but for a different reason.

Given that several mechanisms operate simultaneously, only the joint knowledge of all the mechanisms and how they interact would suffice for taking mechanistic causal claims as a ground for decisions regarding interventions. Unfortunately, the current state of the health sciences is far from this ideal. RCTs and some quasi-experimental research designs are the best sources of evidence for manipulationist causal claims. The hierarchy of evidence, present from the very beginning of the EBM movement (Evidence-Based Medicine Working Group 1992; Wyer and Silva 2009; Djulbegovic and Guyatt 2017), has prioritized this research design over observational studies on humans and animals or in vitro experiments. This prioritization is justified if one focuses on evidence for the narrowly construed interventions that require manipulationist causal claims.

Still, healthcare practice, concerned with all questions regarding diagnosis and therapy, is not and should not be limited to clinical decisions regarding narrowly construed interventions. Other decisions do not require intervening on a cause to influence its effect; hence, justifying them with causal claims invariant under intervention is unnecessary. Among the latter group of clinical decisions, let me distinguish between ‘predicting’ and ‘mechanistic interferences’. While the former type of decision-making can be considered concerned only with making prognoses, predicting, in the broad sense, can be defined as the group of decisions that do not involve modifying the relata of a causal claim. Instead, predicting relies on the use of partial knowledge of a causal structure to act beyond what is represented by that partial causal structure. That is, instead of intervening on (modifying the value of) *X* to influence *Y,* ‘predicting’ involves using the connection between *X* and *Y* to make decisions concerned with some other variable *Z*. For instance, accepting a version of the probabilistic view as one’s epistemic position leads to concluding, based on calculating correlation and studying time precedence, that low barometer readings cause storms (see Cartwright and Efstathiou 2011; Jeffrey 1969). A solution developed by the supporters of the probabilistic approach to causality as an ontological position is to consider separately situations when the common cause is absent or present (Suppes 1970; Otte 1981, 169; Cartwright 1983, 23; Eels 1991, 42). Indeed, if the barometer readings and storms were analyzed separately for cases when air pressure falls and remains constant, the correlation would disappear. For these reasons, as Pearl (1996, 73) observed, “it would make little sense to tamper with a barometer when the goal is to influence the weather”. But the problem re-emerges when one uses a version of the probabilistic theory for causal inference since they are unable to tell with certainty whether a genuine causal relation or a correlation produced by a common cause is discovered.

As Kleinberg (2013, 25) observed, “[i]n biomedical cases, we usually observe only an indicator for the actual event–a measurement of blood pressure, a set of symptoms pointing to heart failure, and so on”. For this reason, despite Cartwright and Efstathiou’s (2011, 227) assertion that it is evident that “you can’t bring on a storm by breaking the glass”, when using observational studies as evidence for clinical decisions, one does not know of whether it is the barometer readings or maybe some other, potentially unobservable variable that causes storms. Nevertheless, some correlations, even if produced by a common cause, might be a ground for some decisions, similar to “barometers [that] remain useful devices to predict the weather” (Wunsch 1986, 2). Clinical decision-making based on observational evidence is connected to accepting some level of uncertainty. At the frontier of science, it remains unknown whether a purported cause is genuinely responsible for the effect or correlational evidence has emerged due to confounding. For example, it remains unknown if paternal age or some characteristic of fathers’ DNA changes the risk of ASD. Therefore, one can either argue, from the perspective of a version of the manipulationist view on causality, that observational studies cannot warrant causal claims or, having in mind the well-being of patients, accept the probabilistic view and consider how probabilistic causal claims can be used in clinical practice. Such claims turn out to help undertake those clinical decisions that do not modify the values of causal claims’ relata. Taking an umbrella when barometer readings are low (with the barometer readings and storms being the relata of the probabilistic causal claim) is likely to be successful as long as the causal structure that produces the observable correlation remains stable despite air pressure being the confounder. In contrast, interventions requiring changing the relata (e.g., pushing the barometer indicator upwards) may fail.

**Fig. 1:**
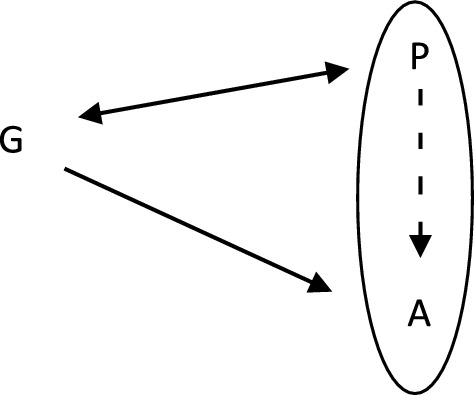
Acting based on knowing partial causal structure (circle indicates the variables and probabilistic dependencies discovered by an epidemiological study, spurious causal claim dotted)

Even though the idea that clinical decisions could be based on the observation of (potentially) spurious correlations produced by some unknown causal structure seems odd at first, physicians undertake them daily while diagnosing patients, making prognoses and prescribing further screening. Such decisions are often informed by a correlation produced by a common cause. Apparently, this is the nature of evidence delivered by the study of Reichenberg et al. (2006). Their conclusions cannot be used to advise men to have children earlier because the research design cannot warrant the claim to be invariant under intervention. Given our understanding of ASD (see Sect. 2.1.), one can hypothesize that paternal age is a proxy for an unobservable variable, such as the number of genetic mutations (*G*) in the male germline. That is, paternal age (*P*) is correlated to genetic mutations *G,* and, because *G* causes (in the manipulationist sense) the prevalence of ASD (*A*), we can observe that *P* probabilistically causes *A* (see Fig. 1). Given this, changing *P* will not change *A,* but we can use this (spurious in the manipulationist sense) causal claim to undertake some clinical decisions. For example, we can assess the psychiatric health of children of older fathers more frequently. Similar to taking umbrellas when a barometer reading is low (and not raising its indicator), this clinical decision can be beneficial despite using probabilistic causal claims describing the potentially spurious causal structure.

Another type of a clinical decision, separate from interventions and predictions, is ‘mechanistic interference’. Such decisions are concerned with either creating an environment for a mechanism to operate in the target of a clinical decision (e.g., a patient) or counteracting a mechanism from working. Like the probabilistic causal claims, mechanistic evidence does not warrant the success of interventions at the aggregate level. Observational studies do not warrant causal claims to be invariant under intervention only if the discovered relations result from spurious correlations. In contrast, even accurate knowledge of one mechanism does not allow for successful interventions because other mechanisms may screen off or even counteract the operation of the mechanism targeted by the mechanistic interference. Given this, the EBM literature locates mechanistic evidence even lower than observational studies on the evidence pyramid. One of the often-cited examples of failed interventions based on mechanistic knowledge is the advice to put infants on their front side (Howick 2011, 154–157). While this advice limits the possibility of choking on one’s vomit (given our current understanding of the phenomenon), some other mechanisms caused infants’ death. For instance, lying on one’s stomach with a face on a pillow may obstruct the respiratory tract. Even though the reliability of mechanistic evidence for decisions concerning narrowly-construed interventions is limited, the EBM hierarchy of evidence underestimates the value of mechanisms. Mechanistic causal claims, which have received the desired attention from the EBM + movement (Parkkinen et al. 2018), can be used for an explanation but also as evidence for ‘mechanistic interferences'.

Creating the mechanism in the target of a clinical decision can be exemplified by intubating patients suffering from acute respiratory distress syndrome (ARDS). The decision regarding mechanical ventilation is not supported by a manipulationist causal claim but by our mechanistic understanding of how blood is oxygenated in the lungs. Counteracting the operation of a mechanism can be instantiated by limiting the exposure of people to artificial blue light based on the mechanistic causal claim of Ratnayake et al. (2018). This interference may not lead to a fall in the prevalence of AMD because we lack difference-making evidence. Such an intervention may stop one of the causes of AMD from operating and improve overall well-being. For example, the mechanistic interference relying on the advice to avoid artificial sources of blue light, which stops the mechanism described by Ratnayake et al. (2018) from operating may not produce observable differences at the populational level because some other mechanisms such as the influence of smoking (Thronton et al. 2005) may account for the number of elders suffering from AMD. In a similar vein, while the advice not to put newborns on their backs has not only failed at reducing the prevalence of infant death syndrome but raised it, the interference has supposedly been effective in stopping the mechanism of how one chokes on their vomit from the operation. Hence, mechanistic evidence can be used to prevent a mechanism from operating or to create a mechanism in the target. However, such interferences do not warrant observable effects at the aggregate level.

Finally, manipulationist causal claims justify undertaking decisions regarding narrowly construed interventions. The random allocation to treatment and control groups in RCTs asserts (in the statistical sense) that the observed difference between outcomes resulted from the intervention. Therefore, if a patient sufficiently resembles the sample admitted to the randomized trial (otherwise, the problem of extrapolation occurs), this evidence warrants that, on average, patients treated with *X* will experience *Y.* For example, the study of Home et al. (2007) delivers evidence that prescribing rosiglitazone to a group of patients resembling the sample of the RECORD trial will bring about better control of glucose levels and raise the prevalence of CHF despite remaining silent on why this effect occurs. Therefore, the manipulationist causal claims warranting invariance under intervention are sufficient grounds for decisions regarding narrowly-construed interventions.

Given this, discovering relations deemed causal by different views on causality is sufficient for undertaking clinical decisions of various types. Predicting can be justified by having observational knowledge of empirical regularities or probabilistic dependencies. While RCTs can deliver such knowledge, their results are not superior to observational studies as grounds for decisions concerning diagnoses and prognoses. Understanding mechanisms is sufficient to conduct mechanistic interferences. Finally, manipulationist causal claims are required for clinical decisions concerned with narrowly-construed interventions.

## Concluding Remarks

Above, we have delivered two arguments for causal pluralism to be endorsed by medical researchers and healthcare practitioners. First, using examples of contemporary medical studies, we argued that the conclusions established on the basis of RCTs, observational epidemiological studies, and laboratory research are best understood as agreeing with, respectively, the manipulationist, probabilistic and mechanistic views on causality. Given this, the pluralistic approach to causality agrees with the whole spectrum of causal inferences in the health sciences. This argument agrees with other studies of inferential practices in medicine that suggest that causal claims are established based on correlational evidence alone. For example, Broadbent (2011) used examples of the harms of alcohol and smoking to illustrate that difference-making evidence can suffice for a causal claim and Vineis and Ghisleni (2004) argued that ethical considerations warrant such a practice in regard to harm.

Second, we have argued that clinical practice is a broad concept encompassing different types of clinical decisions. Each of them requires having at hand specific causal claims. In detail, knowledge of (possibly spurious) empirical regularities is sufficient for acting based on predictions that do not change the relata of causal claims, mechanistic interferences can be conducted based on mechanistic evidence, and the decisions concerned with the narrowly-construed interventions require causal claims that are invariant under intervention. Our second argument develops existing literature supporting pluralism about the concept of cause in medicine. In particular, it develops the position of pragmatic causal pluralism urged by Vandenbroucke et al. (2016) by specifying the types of clinical decisions that can be supported by causal claims understood in line with the three relevant notions of causality.

Further research is needed to address the ontological question of what causation in the domain of medicine actually is. Possibly, there are different types of causal relations that operate in the world. However, the pluralism about the concept of cause, as suggested in the paper, may also be employed by monists regarding the ontology of causation. Even if causation is one type of relation in the world (described by, e.g., a version of the interventionist account, then using different concepts of causality is still warranted if such a practice is beneficial from the pragmatic perspective of gathering useful evidence for specific therapeutic decisions.
